# ADAR activation by inducing a *syn* conformation at guanosine adjacent to an editing site

**DOI:** 10.1093/nar/gkac897

**Published:** 2022-10-16

**Authors:** Erin E Doherty, Agya Karki, Xander E Wilcox, Herra G Mendoza, Aashrita Manjunath, Victorio Jauregui Matos, Andrew J Fisher, Peter A Beal

**Affiliations:** Department of Chemistry, University of California, Davis, CA, USA; Department of Chemistry, University of California, Davis, CA, USA; Department of Chemistry, University of California, Davis, CA, USA; Department of Chemistry, University of California, Davis, CA, USA; Department of Chemistry, University of California, Davis, CA, USA; Department of Chemistry, University of California, Davis, CA, USA; Department of Chemistry, University of California, Davis, CA, USA; Department of Molecular and Cellular Biology, University of California, Davis, CA, USA; Department of Chemistry, University of California, Davis, CA, USA

## Abstract

ADARs (adenosine deaminases acting on RNA) can be directed to sites in the transcriptome by complementary guide strands allowing for the correction of disease-causing mutations at the RNA level. However, ADARs show bias against editing adenosines with a guanosine 5′ nearest neighbor (5′-GA sites), limiting the scope of this approach. Earlier studies suggested this effect arises from a clash in the RNA minor groove involving the 2-amino group of the guanosine adjacent to an editing site. Here we show that nucleosides capable of pairing with guanosine in a *syn* conformation enhance editing for 5′-GA sites. We describe the crystal structure of a fragment of human ADAR2 bound to RNA bearing a G:G pair adjacent to an editing site. The two guanosines form a G_syn_:G_anti_ pair solving the steric problem by flipping the 2-amino group of the guanosine adjacent to the editing site into the major groove. Also, duplexes with 2′-deoxyadenosine and 3-deaza-2′-deoxyadenosine displayed increased editing efficiency, suggesting the formation of a G_syn_:AH^+^_anti_ pair. This was supported by X-ray crystallography of an ADAR complex with RNA bearing a G:3-deaza dA pair. This study shows how non-Watson–Crick pairing in duplex RNA can facilitate ADAR editing enabling the design of next generation guide strands for therapeutic RNA editing.

## INTRODUCTION

ADARs (adenosine deaminases that act on RNA) catalyze adenosine deamination within duplex RNA yielding inosine (1,2). Since inosine is decoded like guanosine, the modification introduced by ADARs can change codon meaning (3). ADARs are multidomain proteins with N-terminal double stranded RNA binding domains (dsRBDs) and C-terminal deaminase domains (4). There are two catalytically active ADARs in humans (ADAR1 and ADAR2) with ADAR1 expressed as two different protein isoforms (p110 and p150) bearing different N-terminal structures (5). Since the substrate for ADARs is an RNA duplex, the enzymes access the reactive adenosine using a base flipping mechanism (6). In addition, the ADAR reaction can be directed to specific adenosines in different transcripts using guide strands that are complementary to these locations because ADARs require duplex RNA for activity. This approach is currently being pursued to develop therapeutic guide strands that recruit ADARs to correct disease-causing mutations in RNA (7–11). While this approach is promising, ADARs have sequence preferences that make certain adenosines disfavored for editing, limiting the current scope of this approach. For instance, the nearest neighbor nucleotide preferences for ADARs show a strong bias against reaction at adenosines with a guanosine 5′ to the target adenosine (5′-GA sites) (12). This preference is explained by our structural studies of ADAR2 bound to transition state analog-containing RNA that suggest a clash between the 2-amino group of the 5′ G and Gly489 of the ADAR2 loop involved in stabilizing the flipped out conformation required for the adenosine deamination reaction (13). Earlier work with fusion proteins bearing ADAR deaminase domains indicated that editing efficiency at 5′-GA sites could be improved with a G:A or G:G pair at the 5′ nearest neighbor (14). However, the basis for this effect has not been reported nor has this effect been established for full length ADARs bearing native dsRBDs. We show here that G:A and G:G pairs on the 5′ side of an editing site improve editing efficiency compared to a 5′ G:C pair for full length ADAR2 and ADAR1 p110. Using X-ray crystallography, we determined the structure of an active fragment of human ADAR2 bound to duplex RNA bearing a G:G pair adjacent to an editing site. The two guanosines form a hydrogen bonded G_syn_:G_anti_ pair and the beneficial effect of this pairing is rationalized by comparison to similar structures with U:A pairs adjacent to ADAR editing sites. In addition, we report the effect on the ADAR deamination rate of several purine analogs paired with a 5′-G in a target site from the *MECP2* transcript where mutation causes Rett Syndrome. Together these results show the use of nucleosides capable of stable pairing with the 5′ G in the *syn* conformation enables editing within 5′-GA target sites.

## MATERIALS AND METHODS

### General biochemical procedures

Molecular-biology-grade bovine serum albumin (BSA) and RNase inhibitor (RNAsin) were purchased from New England BioLabs. SDS-polyacrylamide gels were visualized with a Molecular Dynamics 9400 Typhoon phosphorimager. Data were analyzed with Molecular Dynamics ImageQuant 5.2 software. All Matrix Assisted Laser Desorption/Ionization (MALDI) analyses were performed at the University of California, Davis Mass Spectrometry Facilities using a Bruker UltraFlextreme MALDI TOF/TOF mass spectrometer. Oligonucleotide masses were determined with Mongo Oligo Calculator v2.06. Oligonucleotides for sequencing and PCR were purchased from Integrated DNA Technologies or Dharmacon. All other oligonucleotides were synthesized as described below.

### Synthesis of oligonucleotides

Chemical synthesis for all oligonucleotides was performed using an ABI 394 synthesizer. All protected phosphoramidites were purchased from Glen Research except the 8-azanebularine (azaN) phosphoramidite which was purchased from Berry & Associates or synthesized as previously described (15). Nucleosides were incorporated during the appropriate cycle on a 0.2 or 1.0 μmol scale. Supplementary Tables S1–S5 show sequences of all oligonucleotides used in this study. Upon completion of the synthesis, columns were evaporated under reduced pressure for 12 h. All oligonucleotides were cleaved from the solid support by treatment with 1.5 ml 1:3 ethanol/30% NH_4_OH at 55°C for 12 h. The supernatant was transferred to a new screw-cap tube and evaporated under reduced pressure. For all oligonucleotides except the azaN-modified strand, desilylation was performed by treating the pellets with 250 μl of 1M TBAF–THF at room temperature overnight. For the azaN strand, desilylation was carried out in TEA•3HF as previously described (15). To each reaction was added 75 mM sodium acetate in butanol. The oligonucleotides were then precipitated from a solution of 65% butanol at –70°C for 2 h. The solution was centrifuged at 17 000 × *g* for 20 min, supernatant was removed, and the pellet was washed twice with cold 95% ethanol. The RNA pellets were then desalted using a Sephadex G-25 column and purified as described below.

### Purification of oligonucleotides

Single-stranded RNA oligonucleotides were purified by denaturing polyacrylamide gel electrophoresis and visualized by UV shadowing. Bands were excised from the gel, crushed and soaked overnight at 4°C in 0.5 M NaOAc, 0.1% sodium dodecyl sulfate (SDS), and 0.1 mM EDTA. Polyacrylamide fragments were removed with a 0.2 μm filter, and the RNAs were precipitated from a solution of 75% EtOH at –70°C for 12 h. The solution was centrifuged 17 000 × *g* for 20 min and supernatant was removed. The RNA solutions were lyophilized to dryness, resuspended in nuclease-free water and quantified by absorbance at 260 nm. Oligonucleotide mass was confirmed by MALDI-TOF. Mass spectrometry data can be found in Supplementary Table S6.

### Preparation of duplex RNA substrates for crystallography

For crystallography, the unmodified RNA guide strand was purchased from Horizon Dharmacon and purified as described above. As in previous structures, the edited strand contained the adenosine analog azaN at the editing site. Duplex RNA was hybridized in water in a 1:1 ratio by heating to 95°C for 5 min and slow cooling to 30°C.

### *In vitro* transcription of editing target RNAs

Target RNAs for deamination kinetic analyses were transcribed from DNA templates with the MEGAScript T7 Kit (ThermoFisher). DNA digestion was performed using RQ1 RNase-free DNase (Promega). DNase-treated RNA product was purified as described above.

### Preparation of duplex substrates for ADAR deamination kinetics

Purified guide and transcribed RNA were added in a 10:1 ratio to hybridization buffer (180 nM transcribed RNA target, 1.8 μM guide, 1X TE Buffer, 100 mM NaCl), heated to 95°C for 5 min, and slowly cooled to room temperature.

### Expression and purification of human ADAR2 constructs for deamination kinetics

Full length human ADAR2 (hADAR2) was overexpressed in *Saccharomyces cerevisiae* as previously described (16). Purification of hADAR2 was carried out by lysing cells in buffer containing 20 mM Tris–HCl, pH 8.0, 5% glycerol, 1 mM β-mercaptoethanol (BME), 750 mM NaCl, 35 mM imidazole and 0.01% Nonidet P-40 (NP-40) using a French press. Cell lysate was clarified by centrifugation (39 000 × *g* for 1 h). Lysate was passed over a 3 ml Ni-NTA column, which was then washed in three steps with 20 ml lysis buffer, wash I buffer (20 mM Tris–HCl, pH 8.0, 5% glycerol, 1 mM BME, 750 mM NaCl, 35 mM imidazole, 0.01% NP-40), wash II buffer (20 mM Tris–HCl, pH 8.0, 5% glycerol, 1 mM BME, 35 mM imidazole, 500 mM NaCl), and eluted with 20 mM Tris–HCl, pH 8.0, 5% glycerol, 1 mM BME, 400 mM imidazole, 100 mM NaCl. Fractions containing the target protein were pooled and concentrated to 30–80 μM for use in biochemical assays. Protein concentrations were determined using BSA standards visualized by SYPRO orange staining of SDS-polyacrylamide gels. Purified hADAR2 WT was stored in 20 mM Tris–HCl pH 8.0, 100 mM NaCl, 20% glycerol and 1 mM BME at –70°C.

### Expression and purification of ADAR1 p110 for deamination kinetics

MBP-tagged human ADAR1 p110 construct was cloned into a pSc vector using standard PCR techniques. The generated construct (yeast codon optimized) consisted of an N-terminal MBP-tag, a tobacco etch virus (TEV) protease cleavage site followed by the human ADAR1 p110 gene. *S. cerevisiae* BCY123 cells were transformed with this plasmid and the fusion protein was overexpressed as described previously (17). Purification was carried out by lysing cells in lysis/binding buffer containing 50 mM Tris–HCl, pH 8.0, 5% glycerol, 5 mM BME, 1000 mM KCl, 0.05% NP-40 and 50 μM ZnCl_2_ using a microfluidizer. Cell lysate was clarified by centrifugation (39 000 × *g* for 50 min). Lysate was passed over a 2 ml NEB amylose column (pre-equilibrated with binding buffer), which was then washed in two steps with 50 ml binding buffer followed by 100 ml wash buffer (50 mM Tris–HCl, pH 8.0, 5% glycerol, 5 mM BME, 500 mM KCl, 0.01% NP-40 and 50 μM ZnCl_2_) and eluted with buffer containing 50 mM Tris–HCl, pH 8.0, 10% glycerol, 5 mM BME, 500 mM KCl, 0.01% NP-40, 50 μM ZnCl_2_ and 20 mM maltose. Fractions containing the target protein were pooled and dialyzed against a storage buffer containing 50 mM Tris–HCl, pH 8.0, 400 mM KCl, 0.5 mM EDTA, 0.01% NP-40, 10% glycerol and 1 mM tris(2-carboxyethyl)phosphine. Dialyzed protein was concentrated to 2–50 μM and stored as aliquots at –70°C until further use in biochemical assays. Protein concentrations were determined using BSA standards visualized by SYPRO orange staining of SDS-polyacrylamide gels.

### Deamination assays with ADAR2 and ADAR1 p110

Deamination assays were performed under single-turnover conditions in 15 mM Tris–HCl pH 7.5 3% glycerol, 60 mM KCl, 1.5 mM EDTA, 0.003% NP-40, 3 mM MgCl_2_, 160 U/ml RNAsin, 1.0 μg/ml yeast tRNA, 10 nM RNA, and 75 nM human ADAR2. Each reaction solution was incubated at 30°C for 30 min before the addition of enzyme. Reactions were then incubated at 30°C for varying times prior to quenching with 190 μl 95°C water and heating at 95°C for 5 min. Reaction products were used to generate cDNA using RT-PCR (Promega Access RT-PCR System). DNA was purified using a DNA Clean & Concentrator kit (Zymo) and subjected to Sanger Sequencing via GeneWiz (Azenta). The sequencing peak heights were quantified in SnapGene (Domatics). Data were fit to the equation ${[ P ]_t} = {[ P ]_f}\;[ {1 - {e^{( { - {k_{obs}} \cdot t} )}}} ]$ for ADAR2 where [*P*]_*t*_ is percent edited at time *t*, [*P*]_*f*_ is the final endpoint of editing, and *k_obs_* is the observed rate constant. Because of the slower reactions for ADAR1 p110 and lower reaction end point, data were fit to the equation ${[ P ]_t} = \;0.4 \cdot [ {1 - {e^{( { - {k_{obs}} \cdot t} )}}} ]$. Each experiment was carried out in triplicate where the *k_obs_* reported is the average of each replicate ± standard deviation (SD). Statistical significance between groups was determined by one-way Analysis of Variance (ANOVA) with Tukey's multiple comparisons test using Prism software (GraphPad). For the ADAR1 p110 enzyme, deamination reactions were performed as above with the following modifications: The final reaction solution for ADAR1 p110 contained 15 mM Tris–HCl, pH 7.0 4% glycerol, 26 mM KCl, 40 mM potassium glutamate, 1.5 mM EDTA, 0.003% NP-40, 160 U/ml RNAsin, 1.0 μg/ml yeast tRNA, 10 nM RNA and 250 nM ADAR1 p110.

### Expression and purification of hADAR2 double stranded RNA binding domain and deaminase domain (hADAR2-R2D) for crystallography

Protein expression and purification were carried out by modifying a previously reported protocol (18). *S. cerevisiae* BCY123 cells were transformed with a pSc-ADAR construct encoding hADAR2-R2D E488Q (corresponding to residues 214–701). Cells were streaked on yeast minimal media minus uracil (CM-ura) plates. A single colony was used to inoculate a 15 ml CM-ura starter culture. After cultures were shaken at 300 rpm and 30°C overnight, 10 ml of starter culture was used to inoculate each liter of yeast growth medium. After cells reached an OD_600_ of 1.5 (∼20–24 h) cells were induced with 110 ml of sterile 30% galactose per liter and protein was expressed for 6 h. Cells were collected by centrifugation at 5000 × *g* for 10 min and stored at –80°C. Cells were lysed in 750 mM NaCl in buffer A (20 mM Tris–HCl, pH 8.0, 5% glycerol, 35 mM imidazole, 1 mM BME, and 0.01% Triton X-100) with a microfluidizer. Cell lysate was clarified by centrifugation (39 000 × *g* for 25 min). Lysate was passed over a 5 ml Ni-NTA column equilibrated with buffer A with 750 mM NaCl, which was then washed in three steps with 50 ml of lysis buffer, wash I buffer (buffer A + 300 mM NaCl), and wash II buffer (buffer A + 100 mM NaCl). Protein was eluted with a 35–300 mM imidazole gradient in wash II buffer over 80 min at a flow rate of 1 ml/min. Fractions containing target protein were pooled and further purified on a 2 ml GE Healthcare Lifesciences Hi-Trap Heparin HP column in wash II buffer without BME. The His_10_ fusion protein was washed with 50 ml of wash II buffer without BME and eluted with a 100–1000 mM NaCl gradient over 60 min at a flow rate of 0.8 ml/min. Fractions containing target protein were pooled and cleaved with an optimized ratio of 1 mg of His-tagged TEV protease per 1 mg of protein. Cleavage was carried out for 4 h at room temperature without agitation, followed by overnight cleavage at 4°C before the product was passed over another Ni-NTA column at a flow rate of 0.5 ml/min. The flow through and wash were collected and passed through another Ni-NTA column to remove remaining uncleaved protein. The flow through and wash were collected, dialyzed against 20 mM Tris, pH 8.0, 200 mM NaCl, 5% glycerol and 1 mM BME, followed by concentration to just under 1ml for gel filtration on a GE Healthcare HiLoad 16/600 Superdex 200 PG column. Fractions containing purified protein were pooled and concentrated to 7–9 mg/ml for crystallization trials.

### Crystallization of the hADAR2-R2D E488Q-RNA complex

Crystals of the hADAR2-R2D E488Q-*GLI1* (G:G pair) RNA complex were grown at room temperature by the sitting-drop vapor-diffusion method. A solution of 1.0 μl volume containing 5.6 mg/ml protein (95 μM) and 47.5 μM *GLI1*-GG RNA was mixed with 1.0 μl of 50 mM MOPS pH 7.0, 200 mM NaCl, 17% PEG 4000. Crystals took about 10 days to grow. A cluster of crystals was broken apart and a single cuboid-shaped crystal approximately 100 μm in size was soaked briefly in a solution of mother liquor plus 30% ethylene glycol before flash cooling in liquid nitrogen.

Crystals of the hADAR2-R2D E488Q-*GLI1* (G:3-deaza-dA pair) RNA complex were grown at room temperature by the sitting-drop vapor-diffusion method. A solution of 1.0 μl volume containing 100 μM protein and 50 μM *GLI1*-G3dA RNA was mixed with 1.0 μl of 50 mM MOPS pH 7.0, 100 mM NaCl, and 13% PEG 4000. Flat rhomboid-shape crystals took about 8 days to grow to 100 μm. A single crystal was soaked briefly in a solution of mother liquor plus 30% glycerol before flash cooling in liquid nitrogen.

### Processing and refinement of crystallographic data

X ray diffraction data for both structures were collected at the Advanced Photon Source (APS). Diffraction data for the ADAR2-R2D E488Q *GLI1* (G:G pair) complex were collected on beamline 24-ID-E to 2.7Å resolution while the ADAR2-R2D E488Q *GLI1* (G:3-deaza dA pair) data were collected on the 24-ID-C beamline to 2.8Å resolution. Both data sets were processed with XDS (19) and scaled with AIMLESS (20). The structures were determined by molecular replacement using PHENIX (21). The previous ADAR2-R2D E488Q *GLI1*-bound crystal structure (PDBID: 6vff) was used as the phasing model. The structures were refined with PHENIX including non-crystallographic symmetry (NCS) restraints at the start, but relaxed at the final stages resulting in a lower R-free. Additionally, RNA base-stacking and base-pair restraints, when appropriate, were also imposed in refinement. Table 3 shows statistics in data processing and model refinement.

As observed in the previous ADAR2-R2D-Gli1 structure (22), the asymmetric unit for both structures includes two protein monomers assembled as an asymmetric homodimer complexed with RNA 32 bp duplex. Both complexes displayed similar overall structures. The double stranded RNA binding domains (residues 215–315) of monomer A were disordered and therefore not included in the model. The first 20 residues (215–234) and part of the 5′ RNA binding loop, residues 464–475, of monomer B were disordered and not included in the model. The dsRNA binding domain (dsRBD) of monomer B interacts with the 5′ end of the dsRNA relative to the 8-azanebularine (azaN).

## RESULTS

### A G:G and G:A pair adjacent to an editing site accelerates *in vitro* deamination rates for full length ADAR2 and ADAR1 p110

**Figure 1.**
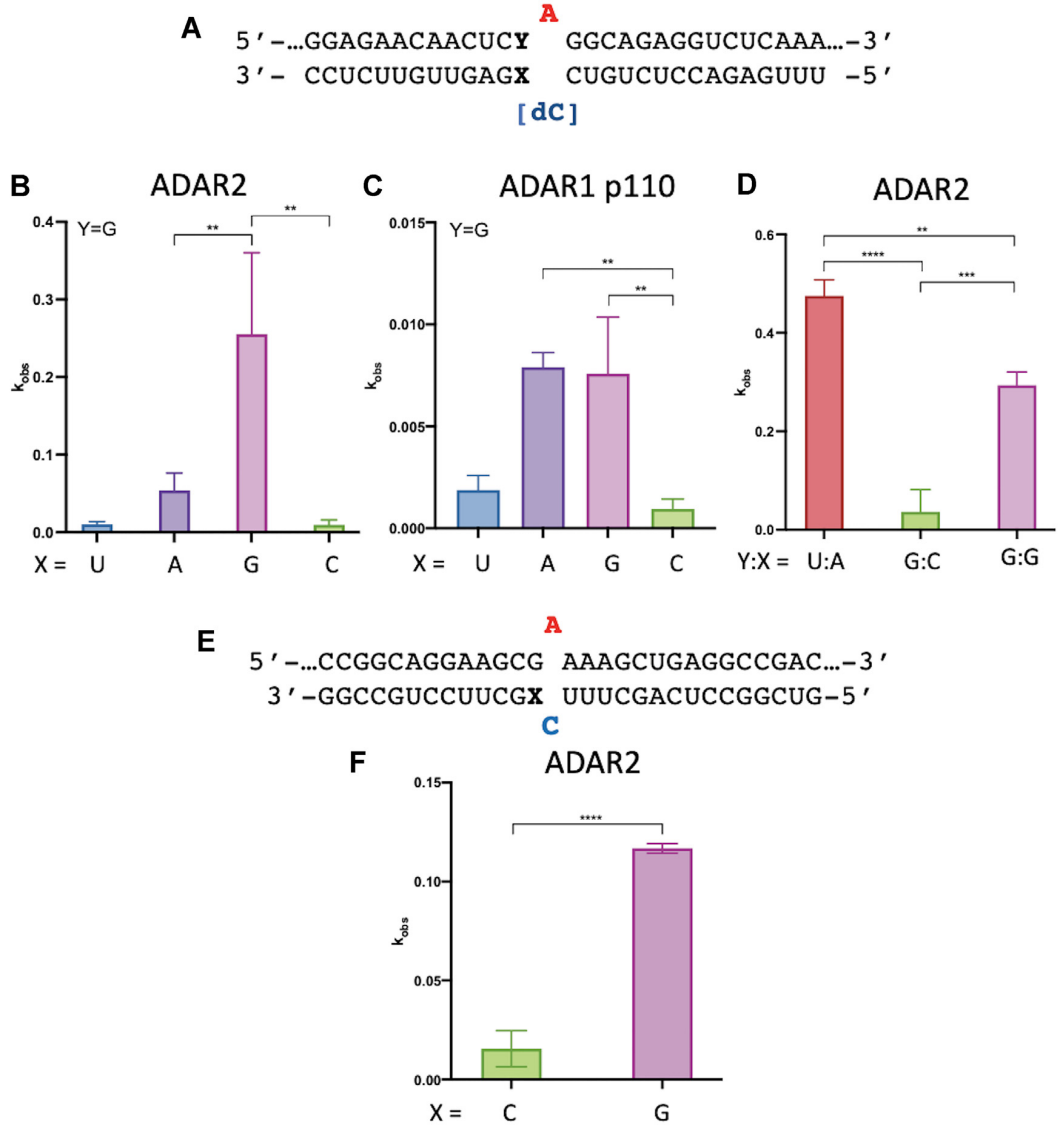
*In vitro* deamination kinetics for ADAR2 and ADAR1 p110 varying 5′ nearest neighbor base pairing. (**A**) Sequence of model substrate for ADARs with varying base pairing adjacent to the editing site (X:Y). The target sequences are derived from the human *IDUA* mRNA. See Table 1 for fitted values. (**B**) Comparison of rate constants for reaction with 100 nM ADAR2. (**C**) Comparison of rate constants with 250 nM ADAR1 p110. (**D**) Comparison of rate constants with 10 nM ADAR2. (**E**) Duplex substrates where target sequence is derived from wild type human *MECP2* mRNA varying base pairing with 5′ G adjacent to the editing site (X). (**F**) Comparison of rate constants for reaction with 100 nM ADAR2. See Table 1 for fitted values. Plotted values are the means of three technical replicates ± standard deviation. Statistical significance between groups was determined using one-way ANOVA with Tukey's multiple comparisons test or an unpaired t-test with Welch's correction; **P < 0.01; ***P < 0.001; ****P < 0.0001.

An earlier report describing optimization of guide strands for SNAP-ADARs, where an ADAR deaminase domain is fused to a SNAP tag and covalently linked guiding oligonucleotide, showed that pairing the 5′ G in a 5′-GAG-codon with either A or G increased editing efficiency compared to a G:C or G:U pair at that site (14). Since this location in an RNA substrate is contacted by the ADAR deaminase domain and not by the dsRBDs, it seemed likely this effect would also be observed with the full length ADARs yet we could find no published account where this was demonstrated. Therefore, we designed model RNA substrates for ADAR1 and ADAR2 where the target editing site is located in a 5′-GA-3′ sequence and the 5′ G base pairing partner was varied (Figure 1A, X = U, A, G or C; Figure 1E, X = C or G). The editing site adenosine was paired with either 2′-deoxycytidine (Figure 1A) or cytidine (Figure 1E). The efficiency of each ADAR reaction was then evaluated by measuring deamination rate constants under single turnover conditions (Tables 1 and 2). As seen with SNAP-ADARs, the G:G pair and G:A pair led to faster adenosine deamination compared to either G:U or G:C. For the sequence shown in Figure 1A, ADAR2 deaminated the substrate with the G:G pair at the fastest rate. However, with ADAR1 p110, the substrates with the G:A pair and G:G pair had similar rates, albeit significantly faster than the G:U and G:C substrates. For comparison, we also measured the rate of the ADAR2 reaction with a similar substrate RNA bearing a 5′ U paired with A, which is the ideal 5′ nearest neighbor base pair (Figure 1D) under conditions where rates for each of these substrates could be measured accurately. While the substrate with the ideal nearest neighbor nucleotides reacts faster, the ADAR2 reaction rate for the 5′ G:G substrate differs by less than two-fold under these conditions (Table 1). Importantly, the rate of deamination of the 5′-G:C substrate was 16-times slower than the ideal substrate under these conditions. The effect of the G:G pair was not limited to a 5′-GAG’-3′ target sequence. The substrate sequence shown in Figure 1E has the target adenosine in a 5′-GAA-3′ sequence and the ADAR2 reaction with 5′-G:G substrate was nearly eight times faster than the 5′-G:C substrate in this sequence context (Table 2). These results confirm the effect of the G:G and G:A pairs in activating editing at 5′-GA sites for the full length ADARs *in vitro*.

**Table 1.** Rate constants for *in vitro* deamination of a model RNA substrate by ADAR2 and ADAR1 p110. ^a^Values for 100 nM ADAR2 acting on 10 nM RNA substrate. ^b^Values for 250 nM ADAR1 p110 acting on 10 nM RNA. ^c^Values for 10 nM ADAR2 acting on 1 nM RNA substrate. Y:X indicates the base pairing adjacent to the editing site. ^d^Data for ADAR2 were fitted to the equation: [*P*]_*t*_ = [*P*]_*f*_[1 – exp(–*k_obs_*·*t*)]. Data for ADAR1 p110 were fitted to the equation: [*P*]*_t_* = 0.4·[1 – exp(–*k_obs_*·*t*)]. ^e^*k_rel_* = *k_obs_* for different nucleosides at Y:X position/*k_obs_* for Y:X = G:C for each of the conditions *a*, *b* and *c*

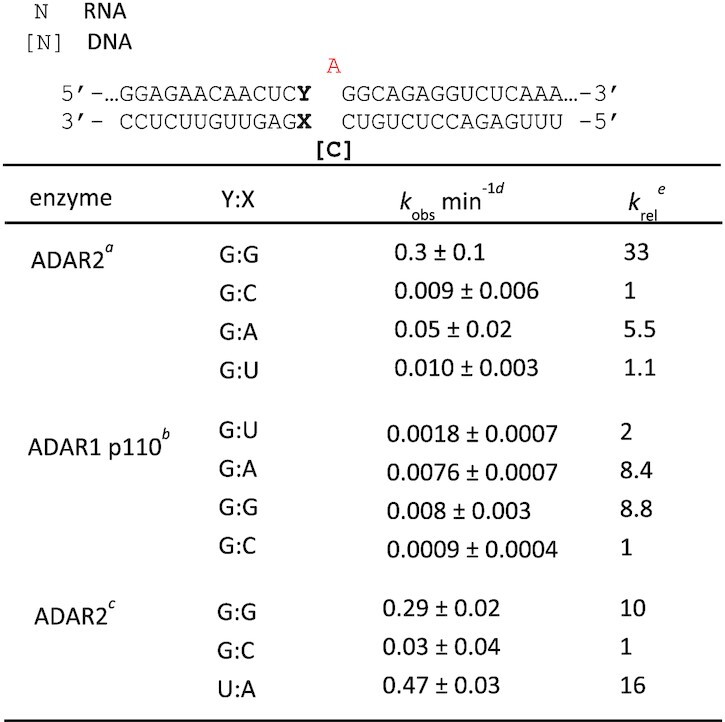

**Table 2.** Rate constants for *in vitro* deamination for 100 nM ADAR2 acting on 10 nM wild type *MECP2* substrate. X indicates the nucleotide in the –1 position. ^a^Data were fitted to the equation: [*P*]_*t*_ = [*P*]_*f*_[1 – exp(–*k_obs_*·*t*)]. ^b^*k_rel_* = *k_obs_* for different nucleosides at X position/*k_obs_* for X = C

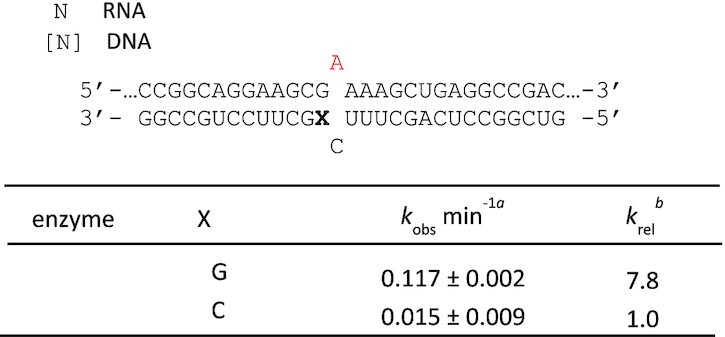

### ADAR2 binds a duplex RNA substrate with a G_(syn)_:G_(anti)_ pair adjacent to the editing site

**Figure 2.**
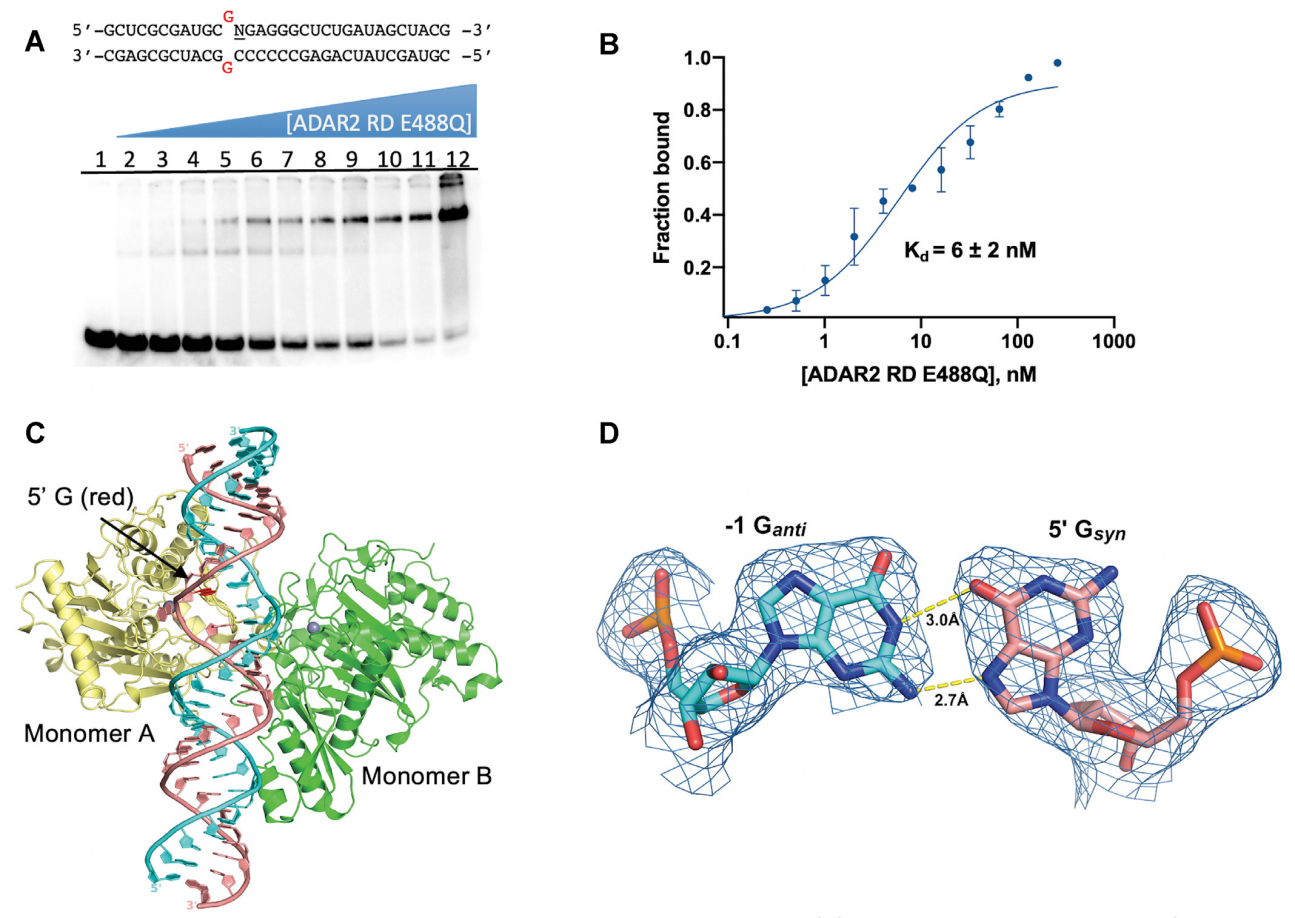
Characterization of complex formed between ADAR2-R2D E488Q and a 32 bp 8-azanebularine (N) containing duplex with G:G pair (32 bp GG RNA) adjacent to N. (**A**) (Top) Sequence of 32 bp duplex used for crystallization. (Bottom) EMSA gel of hADAR2-R2D E488Q with this duplex. Protein concentrations are as follows: lane 1: no protein, lanes 2–12: 0.25, 0.5, 1, 2, 4, 8, 16, 32, 64, 128, 256 nM. (**B**) Quantification of protein binding by EMSA. (**C**) Ribbon diagram depicting the structure of hADAR2-RD E488Q dimer bound to 32 bp G:G pair RNA at 2.7 Å resolution. The edited strand (with azaN flipped into the active site of monomer A is colored salmon, while the unedited guide strand is colored cyan. The 5′G in the *syn* conformation is show with a red base. (**D**) Fit of a G_syn_:G_anti_ base pair in the 2*F*_o_– *F*_c_ electron density map contoured at 1σ. 5′ G indicates guanosine linked to azaN on its 5′ side. –1 G refers to the guanosine in the opposite strand on the 3′ side of the orphan base paired with the 5′ G.

We imagined that a 5′ G:G or 5′ G:A pair could be superior to a 5′ G:C pair for ADAR deamination of the adjacent adenosine in two possible ways. First, since ADARs must distort the duplex RNA and flip the adenosine out of the double helix, a purine:purine pair adjacent to the editing site might destabilize the duplex and facilitate the needed conformational changes. On the other hand, the G:G and G:A combinations may form stable hydrogen bonded pairs whose minor groove structures are more compatible with ADAR binding than that of a Watson–Crick G:C pair. Indeed, several different H-bonded G:G and G:A pairs have been observed in high resolution structures of RNA (23). Knowledge of the nature of the purine:purine interaction could inform the design of other nucleoside analogs for enabling ADAR editing within 5′-GA sequences. Therefore, to gain greater insight into the structural basis for efficient editing by ADAR with a G:G pair adjacent to an editing site, we turned to X-ray crystallography to determine the atomic resolution structure of ADAR2-R2D E488Q bound to the *GLI1* RNA substrate containing the G:G pair. We prepared a 32 bp duplex bearing 8-azanebularine (azaN) at a known editing site for trapping an ADAR-bound complex (Figure 2A). When azaN is properly positioned within an ADAR substrate RNA, ADARs promote its covalent hydration to form a structure that mimics the adenosine deamination intermediate (13,15). We introduced a G:G pair adjacent to the azaN and evaluated its interaction with ADAR2-R2D E488Q. In previous work, we found this combination of ADAR2 E488Q mutant and RNA duplex to be conducive to study by X-ray crystallography (22). ADAR2-R2D E488Q formed a well-defined complex with this duplex as seen in EMSA gels and bound with *K*_d_ = 6 ± 2 nM (Figure 2B). A similar duplex bearing the ideal nearest neighbor (5′-U:A) bound this protein with a *K*_d_ = 0.9 ± 0.5 nM (Supplementary Figure S1).

**Figure 3.**
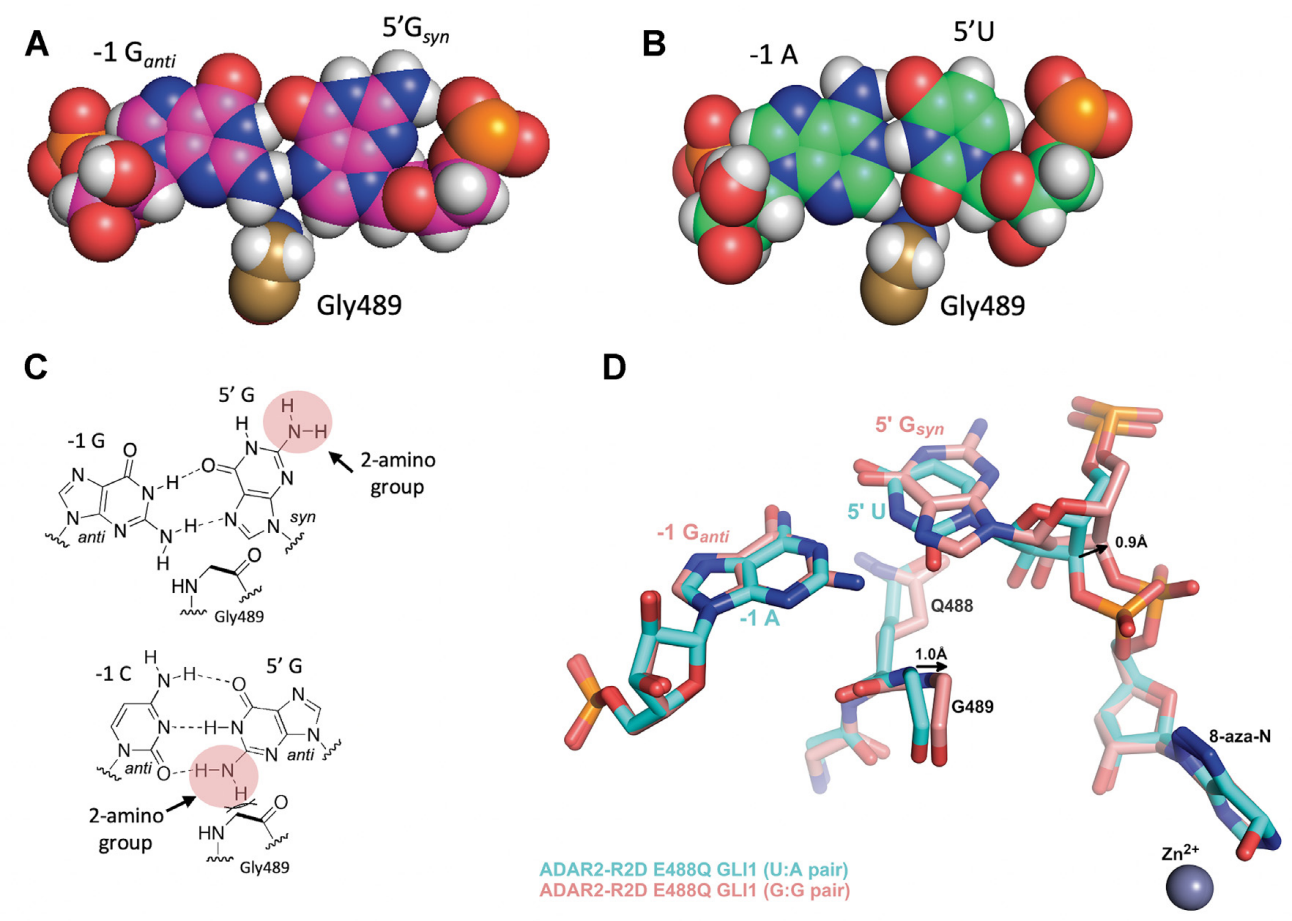
The G_syn_:G_anti_ pair accommodates G489 in the minor groove. (**A**) Space filling representation of the G_syn_:G_anti_ pair and its location relative to G489 in the complex. (**B**) Space filling representation of U:A pair and its location relative to G489 in a complex with ADAR2 for the ideal 5′ nearest neighbor base pair (13). (**C**) (Top) Chemical structure of G_syn_:G_anti_ pair highlighting the location of the 2-amino group of the 5′ G relative to G489. (Bottom) Chemical structure of G_anti_:C_anti_ pair highlighting the location of the 2-amino group of the 5′ G relative to G489. (**D**) Overlay of ADAR2 R2D E488Q structures with RNA bearing either 5′ U paired with A (cyan) or 5′ G paired with G (salmon). Covalent hydration of 8-azanebularine, as seen by the out of plane oxygen present at the C6 position, enables trapping of these structures which mimic the adenosine deamination intermediate as confirmed in previous structures (13).

We were able to grow protein–RNA crystals using the 32 bp azaN-containing duplex with the G:G pairing and ADAR2-R2D E488Q, which diffracted X-rays beyond 2.7 Å resolution (Table 3) (Figure 2C). As we have seen with the related protein–RNA combination (22), the protein bound the RNA as an asymmetric homodimer with the deaminase domain of one monomer (monomer A) involved in direct RNA binding to the flipped-out azaN nucleoside (Figure 2C). Importantly, the G:G pair adjacent to the editing site is well resolved with electron density that best fits a G_syn_:G_anti_ pair with the guanosine on the 5′ side of the azaN in a *syn* conformation with its Hoogsteen face accepting two hydrogen bonds from the Watson–Crick face of the guanosine on the opposing strand (Figures 2D and 3A, Supplementary Figure S2). The guanosine of this guide strand is in an *anti* conformation. The G:G pairing involves N1 to O6 and 2-amino to N7 hydrogen bonding seen in other G_syn_:G_anti_ pairs in RNA (24,25).

**Table 3.** Data processing and refinement statistics for ADAR2-R2D E488Q bound to dsRNA substrates

dsRNA substrate	GLI1 G:G w/azaN 32mer	GLI1 G:3deaza-dA w/azaN 32mer
PDB ID	8e0f	8e4x
Synchrotron (beamline)	APS (24 ID-E)	APS (24 ID-C)
Wavelength (Å)	0.97918	0.97918
Space group	*C*2	*C*2
Unit cell parameters	*a* = 171.52 Å, *b* = 63.39 Å,	*a* = 169.91 Å, *b* = 63.24 Å,
	*c* = 142.13 Å, β = 117.69°	*c* = 142.65 Å, β = 118.07°
Resolution range (Å)	125–2.70 (2.82–2.70)	125–2.80 (2.95–2.80)
No. observed reflections	112 747 (13 563)	99 409 (15 009)
No. unique reflections	36 652 (4467)	31 664 (4741)
Completeness (%)	97.8 (98.6)	95.3 (98.1)
*I*/σ(*I*)	9.6 (1.7)	12.0 (1.2)
*R*_merge_^a^ (%)	7.5 (72.8)	6.4 (117.5)
CC_1/2_	0.995 (0.616)	0.998 (0.524)
**Refinement statistics**		
*R*_factor_^b^ (%)	19.33	19.50
*R*_free_^b^ (%)	23.05	23.94
RMS bond length (Å)	0.007	0.011
RMS bond angle (°)	1.307	1.422
**Ramachandran Plot Statistics** **^c^**		
Favored (%)	96.3	94.5
Allowed (%)	3.0	5.4
Outliers (%)	0.7	0.1
**No. of atoms**		
Protein	6,558	6,558
RNA	1361	1359
Inositol hexakisphosphate (IHP)	72	72
Zn	2	2
Waters	41	42

^a^ *R*_merge_ = [∑_*h*_∑_*i*_|*I*_*h*_ – *I*_*hi*_|/∑_*h*_∑_*i*_*I*_*hi*_] where *I*_*h*_ is the mean of *I*_*hi*_ observations of reflection *h*. Numbers in parenthesis represent highest resolution shell.

^b^ *R*_factor_ and *R*_free_ = ∑||*F*_obs_| – |*F*_calc_|| / ∑|*F*_obs_| × 100 for 95% of recorded data (*R*_factor_) or 5% data (*R*_free_).

^c^Ramachandran plot statistics from MolProbity (38).

The previous structures of ADAR2 bound to RNA revealed the structural basis for ADAR2′s preference for a 5′-U nearest neighbor over 5′-G caused by potential clashes between the 2-amino group and the base-flipping loop residue Gly489 (Figure 3A–C) (13). This minor groove clash can be eliminated for a 5′-G nearest neighbor if the G adopts a *syn* conformation placing the 2-amino group in the major groove (Figure 3A, C). Indeed, data presented here supports this hypothesis with a G:G pair displaying higher editing efficiency and structural evidence affirming the 5′-G *syn* conformation. However, the G_syn_:G_anti_ pair still presents a 2-amino group to the minor groove from the guide strand G_anti_, which causes a slight shift in the base-flipping loop compared to the previous ADAR2-R2D – *GLI1* structure (Figure 3D) (22). The α-carbon of Gly489 shifts ∼1.0 Å towards the edited strand. This shift can be accommodated because of the increased ribose-ribose spacing in a G_syn_:G_anti_ pair as seen in other structures (24,26). Compared to the previous ADAR2-R2D – *GLI1* structure, which contains an A:U pair, the C3′ of the G_syn_ nucleotide also shifts 0.9 Å relative to C3′ of the 5′ U in native *GLI1* RNA (Figure 3D). This shift also causes the phosphate between 5′-G_syn_ and azaN to rotate ∼1.8 Å, however, electron density maps suggest some conformational flexibility in the phosphodiester backbone at this location.

Structural comparisons also identify notable structural features worth mentioning. It is interesting to note that the sugar puckers in both nucleotides of the G_syn_:G_anti_ pair as well as the corresponding A:U pair in native *GLI1* all reside in the 2′-endo conformation. This can be due to the sliding of these bases relative to ideal A-form helical structure, resulting in the widening of the major groove as seen previously (13). Another notable feature is a small difference in how the dsRNA binding domain of monomer B interacts with the RNA. In our original ADAR2-R2D–*GLI1* RNA structure, His259 hydrogen bonds to the 2′OH of C18 of the unedited guide strand, while in this structure, His259 swings over and interacts with the 2′OH of A15 in the edited strand (Supplementary Figure S3). Additionally, Ser258 hydrogen bonds with the 2-amino group of G16 of the edited strand of the previous structure, while in the structure presented here, Ser258 hydrogen bonds with His258 side chain and forms a weak (3.7 Å) interaction with the 2′OH of C19 of the guide strand. A similar conformation is also seen for the G:3-deaza dA pair structure (below). These structural differences together with the weaker electron density of the dsRBDs in all three structures, suggest weak, non-specific interactions between the dsRBDs and the dsRNA.

### Results with nucleoside analogs paired with a 5′ G support the importance of the G_syn_:G_anti_ pair

**Figure 4.**
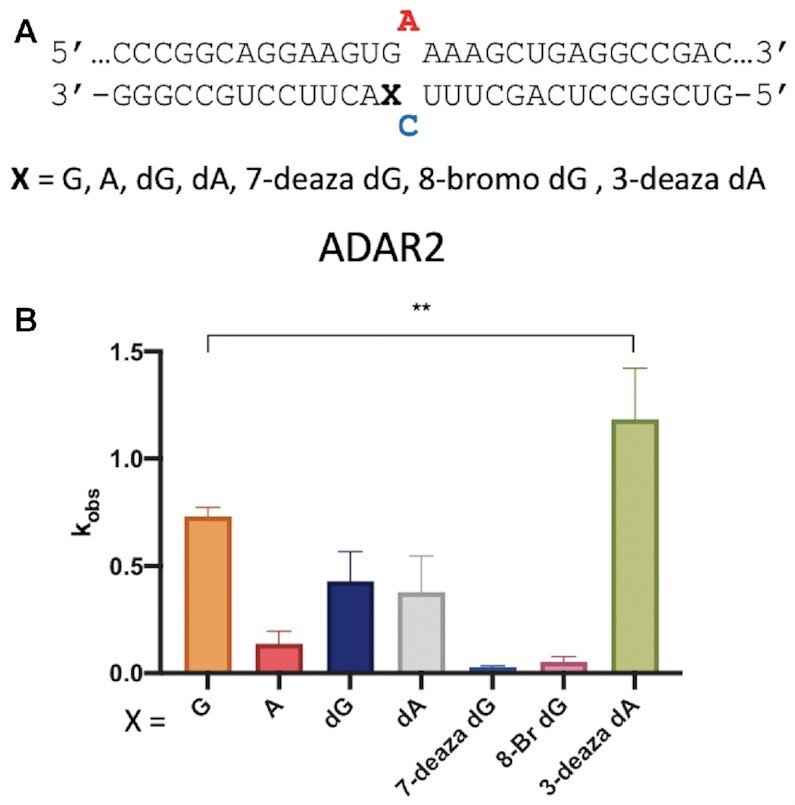
*In vitro* deamination kinetics for ADAR2 and duplex RNAs with nucleoside analogs paired with the 5′ G. (**A**) Sequence of model substrate for ADAR2 derived from the R255X mutant of the human *MECP2* mRNA varying base pairing to the 5′ G adjacent to the editing site (X). X = guanosine (G), adenosine (A), 2′-deoxyguanosine (dG), 2′-deoxyadenosine (dA), 7-deaza-2′-deoxyguanosine (7-deaza dG), 8-bromo-2′-deoxyguanosine (8-bromo dG) and 3-deaza-2′-deoxyadenosine (3-deaza dA) (**B**) Comparison of rate constants for reaction with 100 nM ADAR2. See Table 4 for fitted values. Plotted values are the means of three technical replicates ± standard deviation. Statistical significance between groups was determined using one-way ANOVA with Tukey's multiple comparisons test; **P < 0.01.

The observation of a well-defined G_syn_:G_anti_ pair in the ADAR-RNA complex suggested chemical modifications to the nucleoside paired with the 5′ G might further modulate deamination efficiency. To test this idea, we generated a series of 29 nt ADAR guide RNAs that varied at this position to include several different nucleoside analogs (Figure 4, X = guanosine (G), adenosine (A), 2′-deoxyguanosine (dG), 2′-deoxyadenosine (dA), 7-deaza-2′-deoxyguanosine (7-deaza dG), 8-bromo-2′-deoxyguanosine (8-bromo dG) and 3-deaza-2′-deoxyadenosine (3-deaza dA). The analogs chosen for testing varied in preferred sugar pucker, sterics, and hydrogen-bonding capabilities. The guide RNA sequence was designed to recruit ADARs to induce a potentially corrective edit at the premature termination codon generated by the R255X mutation in the *MECP2* gene associated with Rett Syndrome (Figure 4A) (27). The disease-associated C to T mutation leads to a UGA termination codon in the *MECP2* transcript. While ADAR editing is not capable of restoring the wild type sequence, it can convert the termination codon to one for tryptophan leading to expression of full length R255W mutant MeCP2 protein. We formed RNA duplexes with the modified guides and an RNA transcript bearing the *MECP2* R255X sequence and measured rate constants for deamination at the target adenosine by ADAR2 under single turnover conditions (Table 4). The slow reaction with ADAR1 p110 prevented us from accurately measuring rates for this substrate *in vitro*. As seen with the sequences described above, ADAR2 reacts faster with the substrate bearing a G:G pair compared to G:A pair. The 2′-deoxy modification is well tolerated as both dG and dA support reaction with ADAR2. This is consistent with the 2′-endo sugar pucker observed for G_anti_ found in the G:G pair in the structure described above. The biggest differences in reaction rates were observed with the base-modified purines. Both 7-deaza dG and 8-bromo dG led to very slow deamination reactions. On the other hand, 3-deaza dA paired with the 5′ G led to the fastest ADAR2 reaction measured for the group of analogs tested here.

**Table 4.** Rate constants for *in vitro* deamination for 100 nM ADAR2 acting on 10 nM substrate bearing nucleoside analogs paired with 5′ G (X position). X = guanosine (G), adenosine (A), 2′-deoxyguanosine (dG), 2′-deoxyadenosine (dA), 7-deaza-2′-deoxyguanosine (7-deaza dG), 8-bromo-2′-deoxyguanosine (8-bromo dG) and 3-deaza-2′-deoxyadenosine (3-deaza dA). ^a^Data were fitted to the equation [*P*]_*t*_ = [*P*]_*f*_[1 – exp(–*k_obs_*·*t*)]. ^b^*k_rel_* = *k_obs_* for different nucleosides at X position/*k_obs_* for X = G

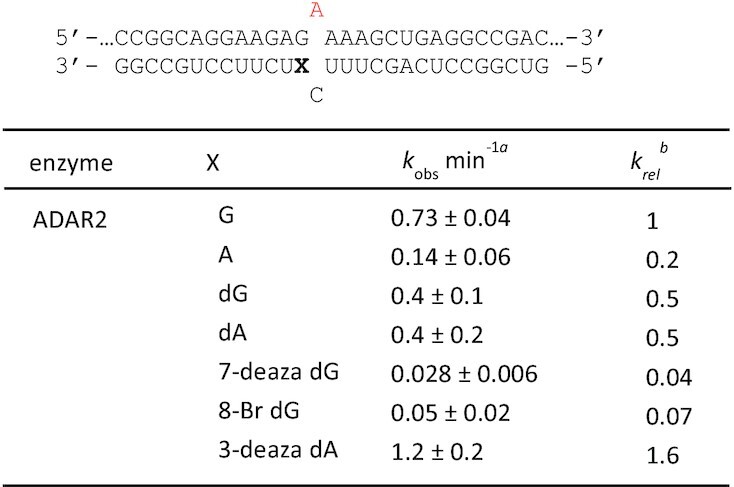

**Figure 5.**
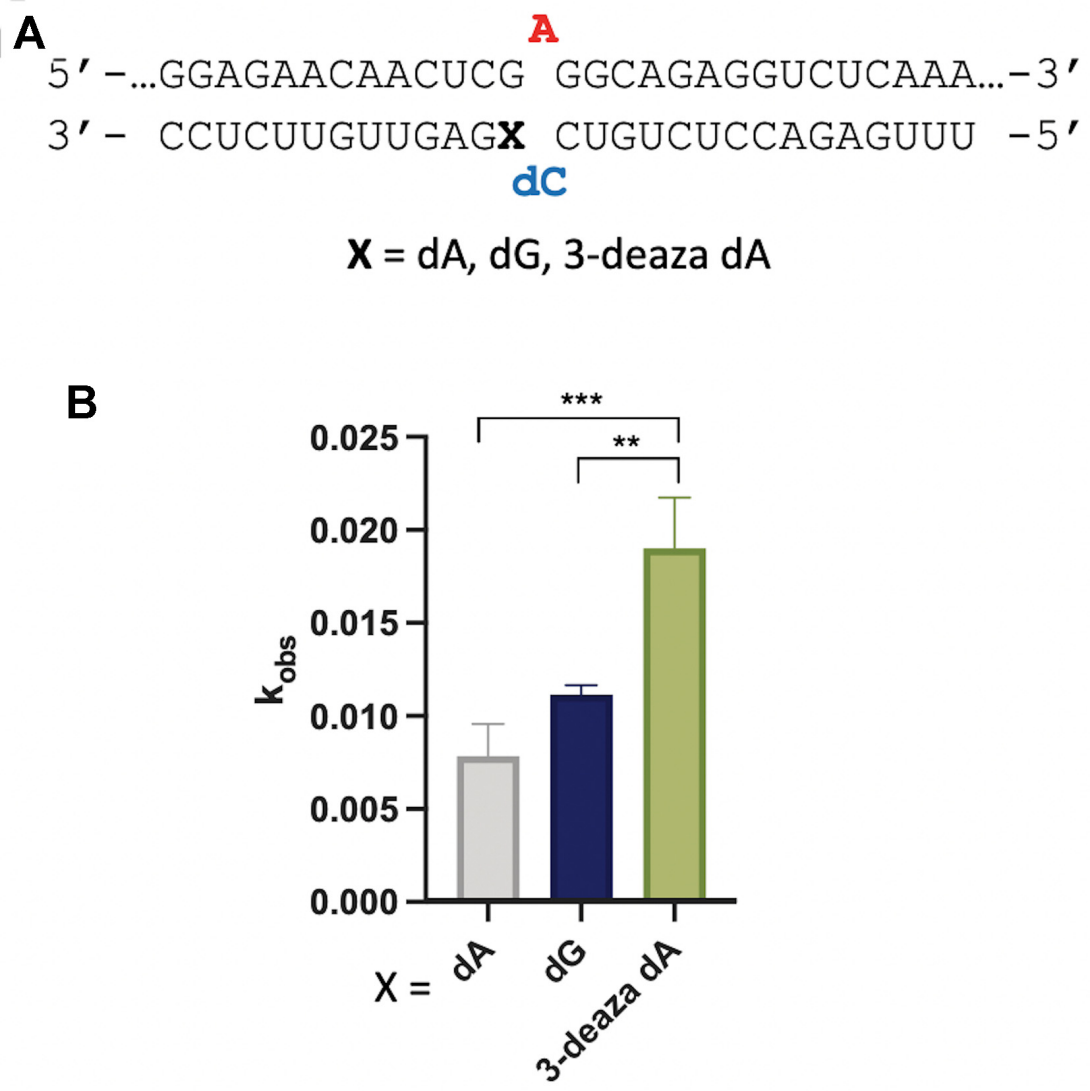
*In vitro* deamination kinetics for ADAR1 p110 and duplex RNAs with nucleoside analogs paired with the 5′ G. (**A**) Sequence of model RNA substrate derived from the human *IDUA* mRNA as in Figure 1A. X = 2′-deoxyguanosine (dG), 2′-deoxyadenosine (dA) and 3-deaza-2′-deoxyadenosine (3-deaza dA) (**B**) Comparison of rate constants for reaction with 250 nM ADAR1 p110. See Table 5 for fitted values. Plotted values are the means of three technical replicates ± standard deviation. Statistical significance between groups was determined using one-way ANOVA with Tukey's multiple comparisons test; **P < 0.01; ***P < 0.001.

While the *MECP2* R255X RNA duplex was a poor substrate for ADAR1 p110 *in vitro*, we were able to measure rates of deamination for this enzyme using the model substrate shown in Figure 1A. Therefore, we introduced the nucleoside analogs dG, dA and 3-deaza dA into the site paired with the 5′ G in that substrate RNA and measured rate constants for deamination by ADAR1 p110 (Figure 5) (Table 5). We found that, like ADAR2, ADAR1 p110 deaminated the substrate bearing the 3-deaza dA paired with the 5′-G at the fastest rate.

**Table 5.** Rate constants for *in vitro* deamination for 250 nM ADAR1 acting on 10 nM substrate bearing nucleoside analogs paired with 5′ G (X position). X = guanosine (G), adenosine (A), 2′-deoxyguanosine (dG), 2′-deoxyadenosine (dA) and 3-deaza-2′-deoxyadenosine (3-deaza dA). ^a^ Data were fitted to the equation [*P*]*_t_* = 0.4·[1 – exp(–*k_obs_*·*t*)]. ^b^*k_rel_* = *k_obs_* for different nucleosides at X position/*k_obs_* for X = G

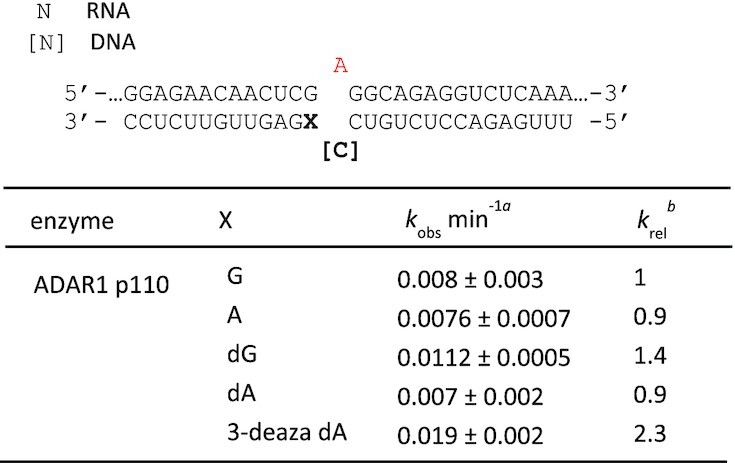

### ADAR2 binds a duplex RNA substrate with a G_(syn)_:AH^+^_(anti)_ pair adjacent to the editing site

**Figure 6.**
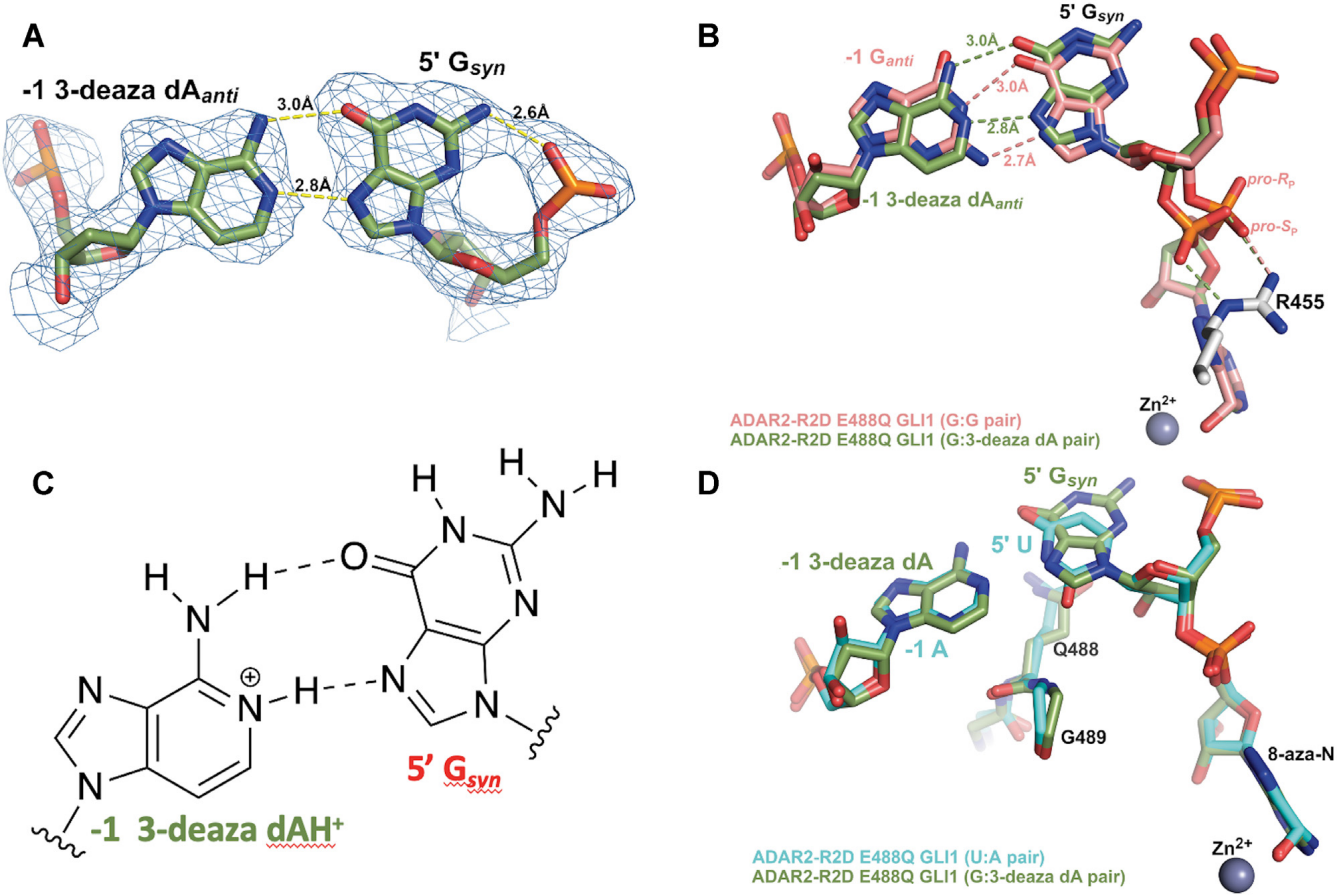
X-ray crystal structure of a complex formed between ADAR2-R2D E488Q and a 32 bp 8-azanebularine (azaN) containing duplex with G:3-deaza dA pair (32 bp G3A RNA) adjacent to azaN. (**A**) Fit of a G_syn_:3-deaza dA_anti_ base pair in the 2*F*_o_– *F*_c_ electron density map contoured at 1σ. (**B**) Overlay of ADAR2 R2D E488Q structures with RNA bearing either 5′ G paired with G (salmon colored carbons) or 5′ G paired with 3-deaza dA (green colored carbons). Arg455 in both structures is identical and shown with white-colored carbons. (**C**) The G_syn_:3-deaza dAH+_anti_ pair (28). (**D**) Overlay of ADAR2 R2D E488Q structures with RNA bearing either 5′ U paired with A (cyan colred carbons) or 5′ G paired with 3-deaza dA (green colored carbons).

The efficient reactions with RNA substrates bearing 3-deaza dA paired with a 5′ G for both ADAR2 and ADAR1 p110 stimulated us to characterize this ADAR-RNA interaction further. Therefore, we prepared a 32 bp duplex bearing azaN adjacent to a 5′ G paired with 3-deaza dA for X-ray crystallography with ADAR2-R2D E488Q (Figure 6). Crystals of this complex formed readily and diffracted X-rays beyond 2.8 Å (Table 3). The overall structure of the asymmetric homodimeric protein bound to RNA is very similar to the structure described above (rmsd of 0.201 Å for 698 equivalent α-carbons) with well-resolved electron density for the purine:purine pair adjacent to the azaN (Figure 6A). The G on the 5′ side of the azaN is in a *syn* conformation with its Hoogsteen face directed toward the Watson–Crick face of the 3-deaza dA on the opposing strand. The position of the substrate strand is shifted slightly from that of the structure described above such that, in this structure, the base pair hydrogen bonding involves 3-deaza dA N1 to G N7 (2.8 Å) and 3-deaza dA 6-amino to G O6 (3.0 Å) (Figure 6B). This interaction causes the base of 3-deaza dA to shift slightly towards the minor groove, while also pushing the 5′G_syn_ out towards the major groove (Figure 6B). This orientation suggests the 3-deaza dA N1 is protonated to donate a hydrogen bond to N7 forming a G_syn_:AH^+^_anti_ pair (28) (Figure 6C). This base pair conformation may be further stabilized by the hydrogen bond between the 2-amino group of the 5′ G_syn_ and its 5′ phosphodiester oxygen with a 2.6 Å distance compared to 3.4 Å in the G_syn_:G_anti_ structure (Figure 6A and B).

Interestingly, the shift in substrate RNA strand position observed in this G_syn_:AH^+^_anti_ structure allows it to adopt a phosphodiester backbone conformation that is nearly identical to that seen in complexes with RNA bearing ADARs’ preferred 5′ nearest neighbor (5′ U paired with A) (Figure 6D). Notably, the phosphate group between the 5′G and azaN swings back towards the guanidino group of Arg455 as seen in all other ADAR2-RNA structures. In the G_syn_:G_anti_ pair structure, only the *pro*-*S*_P_ non-bridging oxygen interacts with Arg455, while in previous ADAR2–RNA complex structures, both non-bridging oxygens interact with the guanidino group of Arg455 (Figure 6B, D). The standard conformation of this phosphate in the G:3-deaza dA pair might contribute to the observed higher deamination rate relative to the G:G pair.

## DISCUSSION

ADARs have a well-established preference for editing adenosines with 5′ nearest neighbor U (or A) and against sites with 5′ nearest neighbor G (12,29,30). This limits the efficiency of therapeutic directed RNA editing applications where the target adenosine has a 5′ G, such as premature UGA termination codons like those arising from the R168X, R255X and R270X mutations in the *MECP2* gene associated with Rett Syndrome (27). In our previously published structures of ADAR2 bound to RNA bearing a 5′ nearest neighbor U, we identified a loop of the protein involved in stabilizing the flipped out conformation (i.e. Ser486-Gly489) that occupied the RNA minor groove spanning three base pairs that included the nearest neighbor nucleotides and the edited base (13,22,31,32). The minor groove edge of the base pair that includes the 5′ nearest neighbor base was juxtaposed to the protein backbone at Gly489. Modeling a G:C pair at this position (i.e. 5′ G) suggested the guanine 2-amino group in the minor groove would clash with the protein at Gly489. In an earlier study, we also showed that replacing a U:A pair at this position with a U-2-aminopurine (2AP) pair resulted in an 80% reduction in deamination rate, further illustrating the detrimental effect of the amino group in the minor groove at this location (13). The G_syn_:G_anti_ pair solves this apparent steric problem by inducing a change in the glycosidic bond angle at the 5′ G from *anti* to *syn*, moving the offending 2-amino group into the major groove where it does not clash with ADAR. G_syn_:G_anti_ base pairs have been observed previously in synthetic RNAs but also in a few biologically relevant cases such as the HIV Rev response element hairpin, the 16S rRNA of the *Escherichia coli* ribosome, and the noncleavable hammerhead ribozyme (26,33–35). In each of these cases and in the case of ADAR2 R2D E488Q bound to an RNA containing a G:G pair the N1 to O6 and 2-amino to N7 hydrogen bonding pattern was observed. This explanation for the effect of the G:G pair on the ADAR reaction seems more likely than the simple effect of a purine:purine mismatch destabilizing the duplex and facilitating base flipping. This is particularly apparent when one considers the fact that the structure of the purine paired with the 5′ G can have a very large effect on the rate enhancement observed. For instance, the substrate with 3-deaza dA paired with a 5′ G reacts with ADAR2 over 40-fold faster than the substrate with 7-deaza dG paired with the 5′ G (Figure 4, Table 4). Thus, the formation of the G_syn_:G_anti_ pair with the editing site 5′ nearest neighbor G in the *syn* conformation explains why the G:G pair on the 5′ side of an editing site enhances ADAR editing compared to G:C or G:U. However, pairing a 5′ G with A also increases the ADAR rate compared to G:C and G:U (Figure 1, Table 1) (14). Importantly, A is also capable of forming a stable pair with G where the G is in a *syn* conformation. When protonated at N1, AH^+^ can donate two hydrogen bonds to the Hoogsteen face of G forming the G_syn_:AH^+^_anti_ pair (Figure 6) (28). The increase in deamination rate observed with 3-deaza dA compared to dA paired with the 5′ G is consistent with the formation of the G_syn_:AH^+^_anti_ pair. The N1H-N7 hydrogen bond of this pair requires protonation of the adenine ring (Figure 6). Since the p*K*_a_ for N1 protonation of 3-deazaadenosine (6.8) is substantially higher than for adenosine (3.7), this site will more likely bear a proton available for hydrogen bonding in the 3-deaza system under the conditions of the ADAR reaction (36). Interestingly, the opposite effect is observed for 7-deaza dG compared to dG. In this case, the higher N1H p*K*_a_ of the 7-deazaguanosine (10.3) compared to guanosine (9.5) weakens the N1H-O6 hydrogen bond in the G_syn_:G_anti_ base pair (36). Indeed, Burkhard and Turner showed that 7-deazaguanosine substitution for either guanosine of a G_syn_:G_anti_ pair is substantially destabilizing in duplex RNA and suggested this was due, at least in part, to the weaker N1H-O6 hydrogen bond formed by 7-deazaguanosine (24). We also found that 8-bromo dG opposite the 5′ nearest neighbor G led to a slow ADAR2 reaction. This was not surprising given the propensity for this nucleoside to adopt a *syn* conformation rendering it incompatible with the type of G_syn_:G_anti_ pair observed in the ADAR complex (37).

In conclusion, the combination of deamination kinetics and structural studies described here identified an approach to facilitate ADAR editing at challenging 5′-GA sites. The use of nucleosides capable of hydrogen bonding to the Hoogsteen face of the 5′ G and inducing a *syn* conformation at this location in the RNA without also introducing additional sterically demanding groups into the minor groove enables efficient editing at these sites.

## DATA AVAILABILITY

Atomic coordinates and structure factors have been deposited in the Protein Data Bank under the accession ID codes: 8e0f, 8e4x.

## Supplementary Material

gkac897_Supplemental_File
