# Supplementary material for: ADAR activation by inducing a syn conformation at guanosine adjacent to an editing site
Source: Nucleic Acids Res. 2022 Oct 16;50(19):10857–68. doi: 10.1093/nar/gkac897 (PMC9638939; doi:10.1093/nar/gkac897)
Supplement: gkac897_Supplemental_File [file gkac897_supplemental_file.pdf]

Supporting Information

ADAR Activation by Inducing a Syn Conformation at Guanosine Adjacent to an Editing Site

Erin E. Doherty[1], Agya Karki[1], Xander E. Wilcox[1], Herra G. Mendoza[1], Aashrita Manjunath[1],Victorio Jauregui Matos [1], Andrew J. Fisher*[1,2] and Peter A. Beal*[1]

[1]Department of Chemistry, University of California, Davis, CA, USA.

[2]Department of Molecular and Cellular Biology, University of California, Davis, CA, USA.

*Correspondence to: ajfisher@ucdavis.edu, pabeal@ucdavis.edu

Contents

Table S1 Guide sequences for *in vitro* kinetics of the *IDUA* target 2

DNA template sequence for *in vitro* kinetics of the *IDUA* 5'-UA target 2

DNA template sequence for *in vitro* kinetics of the *IDUA* 5'-GA target 2

Table S2 Guide sequences for *in vitro* kinetics of the *MECP2* R255 target 3

DNA template sequence for *in vitro* kinetics of the *MECP2* R255 target 3

Table S3 Sequences for *in vitro* kinetics of the *MECP2* R255X target 3-4

DNA template sequence for *in vitro* kinetics of the *MECP2* R255X target 4

Table S4 Oligonucleotide sequences for crystallography. 4

Table S5 Oligonucleotide sequences for ADAR binding experiments. 4

Conditions for gel mobility shift assay of duplex RNA and ADAR2 R2D E488Q 4-5

Supplementary Figure S1 5

Supplementary Figure S2 6

Table S6 Oligonucleotide mass spectrometry data 7

Supplementary Figure S3 8

Table S1 Sequences for *in vitro* kinetics of the *IDUA* target (nucleotides in brackets are 2'-deoxy. All others are ribonucleotides). All PCR primers are 2'-deoxynucleotides. (3dA) indicates 3-deaza-2'-deoxyadenosine.

IDUA guide strand -1 A	5'-UUUGAGACCUCUGUC[C]AGAGUUGUUCUCC-3'
IDUA guide strand -1 G	5'-UUUGAGACCUCUGUC[C]GGAGUUGUUCUCC-3'
IDUA guide strand -1 C	5'-UUUGAGACCUCUGUC[C]CGAGUUGUUCUCC-3'
IDUA guide strand -1 U	5'-UUUGAGACCUCUGUC[C]UGAGUUGUUCUCC-3'
IDUA guide strand -1 2'-deoxy G	5'-UUUGAGACCUCUGUC[CG]GAGUUGUUCUCC-3'
IDUA guide strand -1 2'-deoxy A	5'-UUUGAGACCUCUGUC[CA]GAGUUGUUCUCC-3'
IDUA guide strand -1 2'-deoxy-3-deazaadenosine	5'-UUUGAGACCUCUGUC[C(3dA)]GAGUUGUUCUCC-3'
Idua RT-PCR forward and sequencing primer	5'-GCTCCTCCCATCCTGTGGGCTGAACAGT-3'
Idua RT-PCR reverse primer	5'-CGGGGTGTGCGTGGGTGTCATCACT-3'

DNA template sequence for *in vitro* kinetics of the *IDUA* 5'-UA target. Grey indicates the T7 promoter, underline is the region complementary to the guide strands, and the red A is the target adenosine.

TAATACGACTCACTATAGGGCTCCTCCCATCCTGTGGGCTGAACAGTATAACAGACT
CCCAGTATACAAATGGTGGGAGCTAGATATTAGGGTAGGAAGCCAGATGCTAGGTA
TGAGAGAGCCAACAGCCTCAGCCCTCTGCTTGGCTTATAGATGGAGAACAACTCTAG
GCAGAGGTCTCAAAGGCTGGGGCTGTGTTGGACAGCAATCATACAGTGGGTGTCCT
GGCCAGCACCCATCACCCTGAAGGCTCCGCAGCGGCCTGGAGTACCACAGTCCTCA
TCTACACTAGTGATGACACCCACGCACACCCCGGATCC

DNA template sequence for *in vitro* kinetics of the *IDUA* 5'-GA target. Grey indicates the T7 promoter, underline is the region complementary to the guide strands, and the red A is the target adenosine.

TAATACGACTCACTATAGGGCTCCTCCCATCCTGTGGGCTGAACAGTATAACAGACT
CCCAGTATACAAATGGTGGGAGCTAGATATTAGGGTAGGAAGCCAGATGCTAGGTA
TGAGAGAGCCAACAGCCTCAGCCCTCTGCTTGGCTTATAGATGGAGAACAACTCGA
GGCAGAGGTCTCAAAGGCTGGGGCTGTGTTGGACAGCAATCATACAGTGGGTGTCC
TGGCCAGCACCCATCACCCTGAAGGCTCCGCAGCGGCCTGGAGTACCACAGTCCTC
ATCTACACTAGTGATGACACCCACGCACACCCCGGATCC

Table S2 Sequences for *in vitro* kinetics of the *MECP2* R255 target. All guides are ribonucleotides. All PCR primers are 2'-deoxynucleotides.

MECP2 R255 guide strand -1 C	5'-GUCGGCCUCAGCUUUCCGCUUCCUGCCGG-3'
MECP2 R255 guide strand -1 G	5'-GUCGGCCUCAGCUUUCGGCUUCCUGCCGG-3'
MECP2 R255 RT-PCR forward and sequencing primer	5'-GTGCAGGTGAAAAGGGTC-3'
MECP2 R255 RT-PCR reverse primer	5'-TACGGTCTCCTGCACAGATCG-3'

DNA template sequence for *in vitro* kinetics of the *MECP2* R255 target. Grey indicates the T7 promoter, underline is the region complementary to the guide strands, and the red A is the target adenosine.

TAATACGACTCACTATAGGGGTGCAGGTGAAAAGGGTCCTGGAGAAAAGTCCTGGG
AAGCTCCTTGTCAAGATGCCTTTTCAAACTTCGCCAGGGGGCAAGGCTGAGGGGGGT
GGGGCCACCACATCCACCCAGGTCATGGTGATCAAACGCCCCGGCAGGAAGCGAAA
AGCTGAGGCCGACCCTCAGGCCATTCCCAAGAAACGGGGCCGAAAGCCGGGGAGTG
TGGTGGCAGCCGCTGCCGCCGAGGCCAAAAAGAAAGCCGTGAAGGAGTCTTCTATC
CGATCTGTGCAGGAGACCGTA

Table S3 Sequences for *in vitro* kinetics of the *MECP2* R255X target. Nucleotides in brackets are 2'-deoxy. All others are ribonucleotides. All PCR primers are 2'-deoxynucleotides. (8BrG) is 8-bromo-2'-deoxyguanosine, (7dA) is 7-deazaadenosine, (3dA) is 3-deazaadenosine

MECP2 R255X guide strand -1 C	5'-GUCGGCCUCAGCUUUCCACUUCCUGCCGG -3'
MECP2 R255X guide strand -1 G	5'-GUCGGCCUCAGCUUUCGACUUCCUGCCGG -3'
MECP2 R255X guide strand -1 A	5'-GUCGGCCUCAGCUUUCAACUUCCUGCCGG -3'
MECP2 R255X guide strand -1 2'-deoxy A	5'-GUCGGCCUCAGCUUUC[A]ACUUCCUGCCGG -3'
MECP2 R255X guide strand -1 2'-deoxy G	5'-GUCGGCCUCAGCUUUC[G]ACUUCCUGCCGG -3'
MECP2 R255X guide strand -1 2'-deoxy 8-bromoguanosine	5'-GUCGGCCUCAGCUUUC[(8BrG)]ACUUCCUGCCGG -3'
MECP2 R255X guide strand -1 2'-deoxy 7-deazaguanosine	5'-GUCGGCCUCAGCUUUC[(7dG)]ACUUCCUGCCGG -3'
MECP2 R255X guide strand -1 2'-deoxy 3-deazaadenosine	5'-GUCGGCCUCAGCUUUC[(3dA)]ACUUCCUGCCGG -3'

MECP2 R255X RT-PCR forward and sequencing primer	5'-GGGTGTGCAGGTGAAAAGG-3'
MECP2 R255X RT-PCR reverse primer	5'-TCTTGATGGGGAGTACGGTC-3'

DNA template sequence for *in vitro* kinetics of the *MECP2* R255X target. Grey indicates the T7 promoter, underline is the region complementary to the guide strands, and the red A is the target adenosine.
CACGATTAATACGACTCACTATAGGGTGTGCAGGTGAAAAGGGTCCTGGAGAAAAG
TCCTGGGAAGCTCCTTGTCAAGATGCCTTTTCAAACTTCGCCAGGGGGCAAGGCTGA
GGGGGGTGGGGCCACCACATCCACCCAGGTCATGGTGATCAAACGCCCCGGCAGGA
AGTGAAAAGCTGAGGCCGACCCTCAGGCCATTCCCAAGAAACGGGGCCGAAAGCCG
GGGAGTGTGGTGGCAGCCGCTGCCGCCGAGGCCAAAAAGAAAGCCGTGAAGGAGT
CTTCTATCCGATCTGTGCAGGAGACCGTACTCCCCATCAAGAA

Table S4 Sequences for crystallography. All bases are ribonucleotides. (N) is 8-azanebularine.

GLI1(GG, G3dA) 32mer top with 8-azanebularine	5'- GCUCGCGAUGCG(N)GAGGGCUCUGAUAGCUACG -3'
GLI1 (GG) 32mer bottom	5'- CGUAGCUAUCAGAGCCCCCCGGCAUCGCGAGC -3'
GLI1 (G3dA) 32mer bottom	5'- CGUAGCUAUCAGAGCCCCCC(3dA)GCAUCGCGAGC -3'

Table S5 Sequences for binding experiments. All bases are ribonucleotides. (N) is 8-azanebularine.

GLI1(GG) 32mer top with 8-azanebularine	5'- GCUCGCGAUGCG(N)GAGGGCUCUGAUAGCUACG -3'
GLI1 (GG) 32mer bottom	5'- CGUAGCUAUCAGAGCCCCCCGGCAUCGCGAGC -3'
GLI1(UA) 32mer top with 8-azanebularine	5'- GCUCGCGAUGCU(N)GAGGGCUCUGAUAGCUACG -3'
GLI1 (UA) 32mer bottom	5'- CGUAGCUAUCAGAGCCCCCCAGCAUCGCGAGC-3'

Conditions for gel mobility shift assay of duplex RNA and ADAR2-R2D E488Q

The 5'-end of top strand containing 8-azanebularine was labeled using [γ-^{32}P] ATP (6000Ci/mmol) and NEB polynucleotide kinase. The labeled reaction was passed through a G-25 column to remove excess ATP and further purified with a 19% denaturing page gel at 10 W for 8 h. The labeled oligonucleotide was visualized by storage phosphor autoradiography and the gel band containing it was excised, crushed, soaked and ethanol precipitated as described for other gel-purified oligonucleotides used in this study. The labeled strand was diluted to a stock solution of approximately 300 nM and hybridized to its compliment at 1:3 ratio in 1X TE buffer, pH 7.5 and 200 mM NaCl by heating at 95 °C for 5 min and slowly cooling to 30 °C to a final concentration

of approximately 50 nM. Samples containing ≤ 1 nM RNA and varying concentrations of the protein (260, 130, 65, 32.5, 16.2, 8.1, 4.1, 2.0, 1.0, 0.5, 0.25 and 0 nM) were incubated together in 20 mM Tris-HCl, pH 7.0, 3.5% glycerol, 0.5 mM DTT, 60 mM KCl, 20 mM NaCl, 0.1 mM β-mercaptoethanol, 1.5 mM EDTA, 0.003% Nonidet P-40, 160 units/ul RNase inhibitor, 100 μg/mL BSA, and 1 μg/mL yeast tRNA for 30 min at 30 °C. Samples were loaded onto a 6% gel, electrophoresed in nondenaturing polyacrylamide gel (79:1 acrylamide:bisacrylamide) in 1× TBE buffer at 4 °C for 90 min. The gels were dried on a Biorad gel drier for 90 min at 80 °C under vacuum followed by exposure to storage phosphor imaging plates (Kodak) for 24 h in the dark. After exposure, the gels were removed, and the phosphor imaging plates were scanned by Typhoon Trio Variable Mode Imager (GE Healthcare). Dissociation constants were measured by calculating the fraction of RNA bound by the protein and using the equation fraction bound = A*([protein]/[protein]+K_d), where the K_d is the fitted apparent disassociation constant and A is the fitted maximum fraction of RNA bound. Specific 32mer sequences used in the assay are described in the supplementary table.

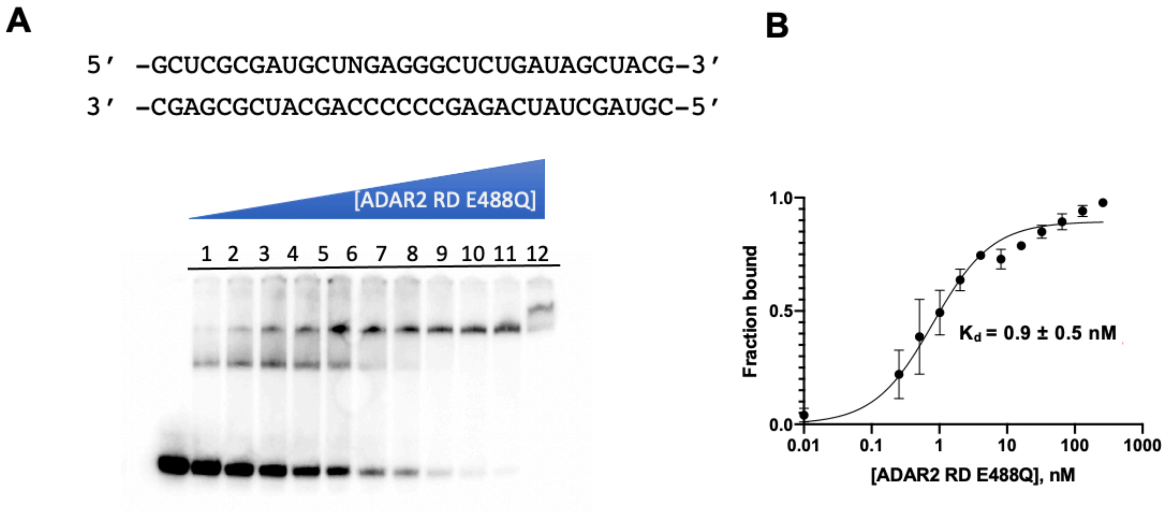

Supplementary Figure S1: **(A)** Representative electrophoretic mobility shift assay (EMSA) gel displaying tightly bound complex of ADAR2-R2D E488Q to 8-azanebularine (N)-containing hGLI1 32 bp (5'U) duplex. Lane 1: no protein added; Lanes 2-12: 0-260 nM protein. **(B)** Fitted plot of fraction RNA bound vs. ADAR2-R2D E488Q concentration.

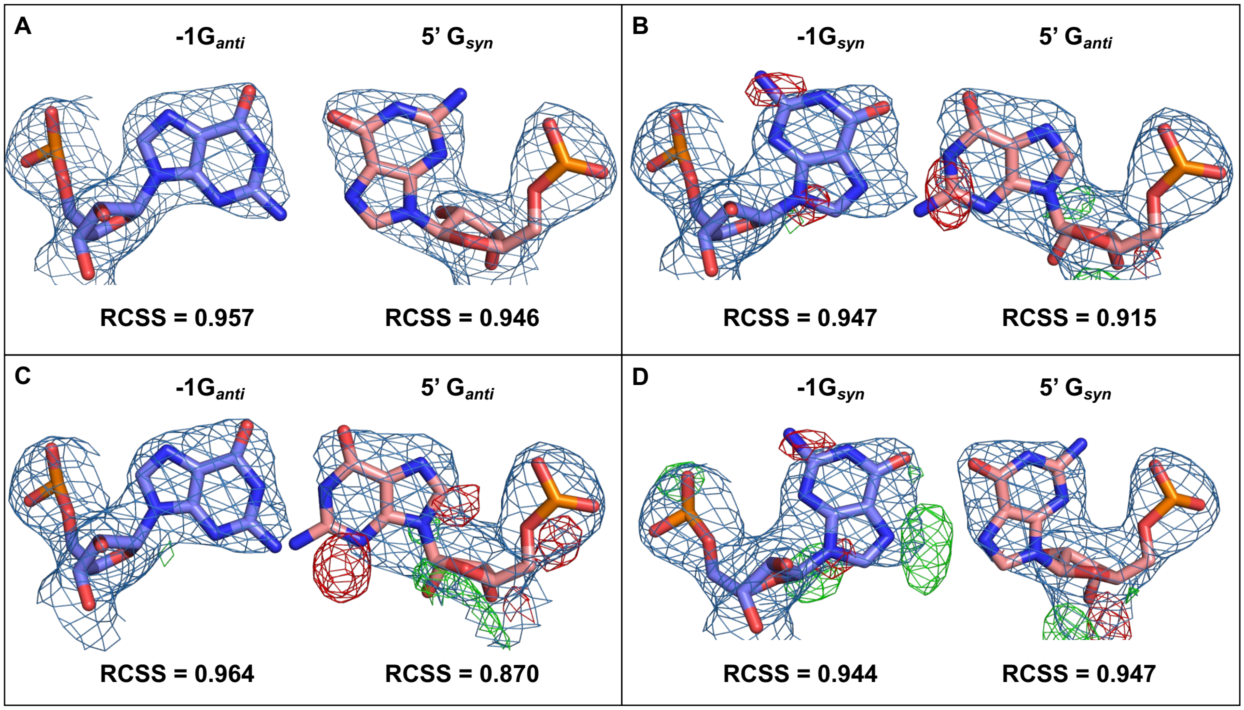

Supplementary Figure S2: **Analysis of the Fo-Fc and 2Fo-Fc maps of all possible conformations for the 5' and -1 positions with corresponding Real Space Correlation Coefficients (RCSS).** To determine to correct conformations of the adjacent guanosines, each anti-conformation and syn-conformation permutation was modeled where the 5'G position refers to the guanosine 5' of the 8-azanebularine and the -1G refers to the -1 position relative to the orphan base. Once each combination was modeled, the model was refined, and the F_o-F_c (green and red, positive and negative electron density, respectively) and $2F_o$-F_c (blue) maps were analyzed. Additionally, the real space correlation coefficients (RCSS) for each guanosine were calculated in PHENIX to determine which conformations fit the density the best. **(A)** The guanosine at the -1 position is modeled in the *anti*-conformation whereas the guanosine at the 5' position is modeled in the *syn*-conformation. **(B)** The guanosine at position -1 modeled as *syn* and the G at the 5' position modeled as *anti*. **(C)** Both guanosines at the -1 and 5' positions modeled in the *anti*-conformation. **(D)** The guanosines at both the -1 and 5' positions modeled in the *syn* conformations.

Table S6 Oligonucleotide mass spectrometry data.

Guide	Calculated (g/mol)	Observed (g/mol)
IDUA guide strand -1 A	9118.4	9118.6
IDUA guide strand -1 C	9096.4	9096.8
IDUA guide strand -1 U	9096.4	9094.8
IDUA guide strand -1 G	9135.4	9132.1
IDUA guide strand -1 2'-deoxy G	9116.1	9121.9
IDUA guide strand -1 2'-deoxy A	9100.1	9092.8
IDUA guide strand -1 2'-deoxy-3-deaza adenosine	9099.1	9089.7
MECP2 R255 guide strand -1 C	9137.1	9134.3
MECP2 R255 guide strand -1 G	9177.1	9173.5
MECP2 R255X guide strand -1 G	9161.2	9164.4
MECP2 R255X guide strand -1 A	9145.2	9150.1
MECP2 R255X guide strand -1 2'-deoxy G	9145.2	9155.6
MECP2 R255X guide strand -1 2'-deoxy A	9129.2	9140.2
MECP2 R255X guide strand -1 2'-deoxy 7-deaza guanosine	9144.2	9130.7
MECP2 R255X guide strand -1 2'-deoxy 8-bromo guanosine	9225.1	9235.4
MECP2 R255X guide strand -1 2'-deoxy 3-deaza adenosine	9128.2	9138.7
GLI1 32mer top with 8-azanebularine	10328.3	10329.1
GLI1(GG) 32mer bottom	10206.4	10202.7
GLI1 (G3dA) 32mer bottom	10173.4	10179.6

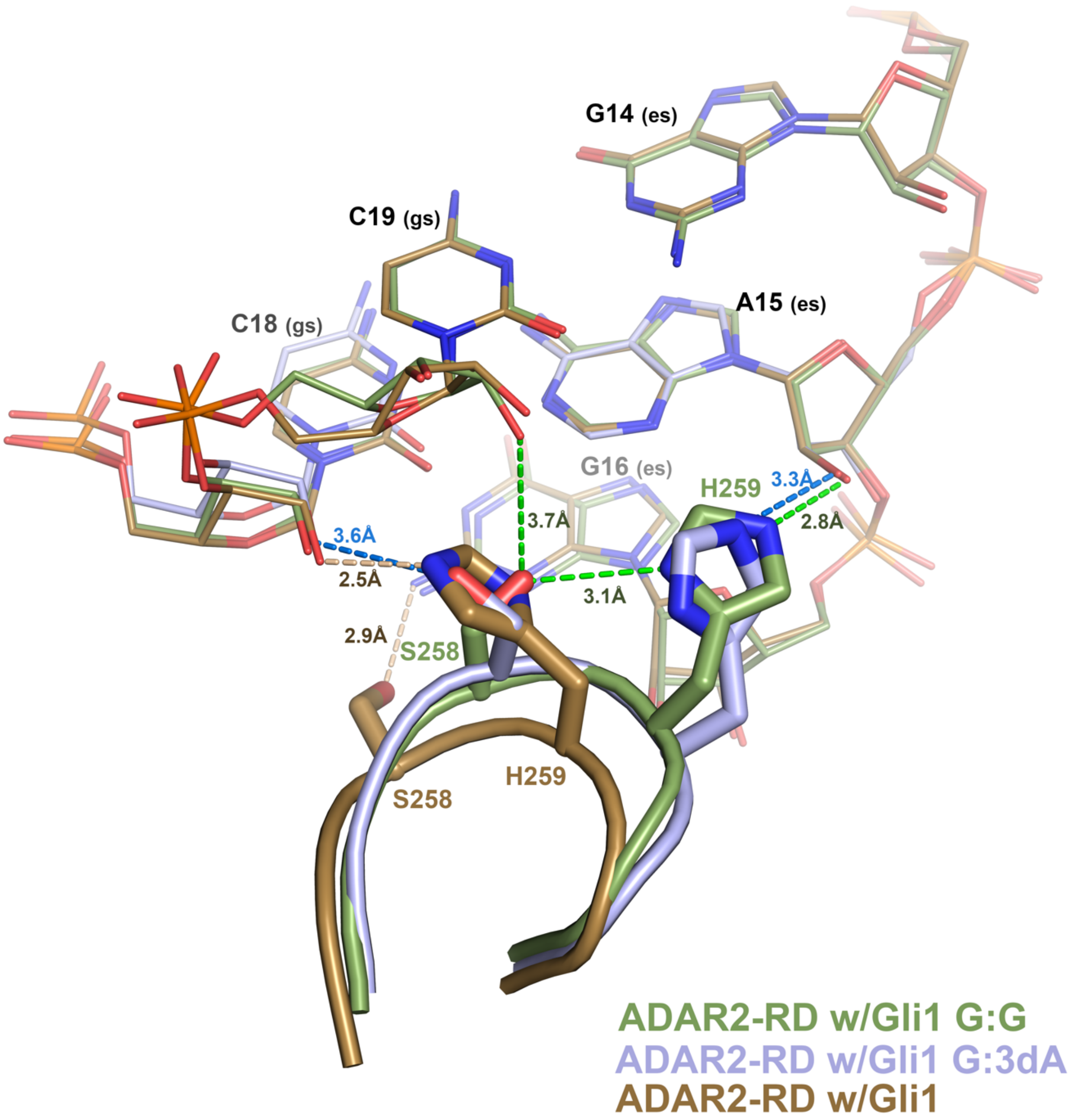

Supplementary Figure S3: **Comparison of ADAR2's dsRBD2 interactions with RNA.** Shown is the superposition of ADAR2-R2D E488Q – Gli1 G:G pair (green) and ADAR2-R2D E488Q – Gli1 G:3-deaza-dA pair (light blue), with our previously reported structure of ADAR2-R2D E488Q – Gli1 (PDBID 6vff) (in sand color). Note the different RNA contacts involving residues Ser258 and His259 between the three structures. For clarity, only the A15(es) and C18(gs) bases for the G:3-deaza-dA pair are shown. Dashed lines display potential hydrogen bonds with the colors corresponding to the respective structures. es = edited strand, gs = guide strand.
